# Supplementary material for: Rural–Urban Differences in Dietary Behavior and Obesity: Results of the Riskesdas Study in 10–18-Year-Old Indonesian Children and Adolescents
Source: Nutrients. 2019 Nov 18;11(11):2813. doi: 10.3390/nu11112813 (PMC6893820; doi:10.3390/nu11112813)
Supplement: Supplementary file 1 [file nutrients-11-02813-s001.pdf]

Table S1. Body mass index (BMI) per age for Indonesian populations based on gender

Age	Boys				Girls			
	<5th percentile	5th - <85th percentile	85th - < 95th percentile	≥ 95th percentile	<5th percentile	5th - <85th percentile	85th - < 95th percentile	≥ 95th percentile
	Underweight (BMI <)	Healthy weight (≥BMI<)	Overweight (≥BMI<)	Obesity (BMI ≥)	Underweight (BMI <)	Healthy weight (≥BMI<)	Overweight (≥BMI<)	Obesity (BMI ≥)
10	12.9	12.9-19.9	19.9-23.7	23.7	12.8	12.8-19.7	19.7-23.2	23.2
11	12.8	12.8-20.6	20.6-24.4	24.4	12.8	12.8-20.5	20.5-23.7	23.7
12	12.9	12.9-20.7	20.7-24.4	24.3	13.3	13.3-21.0	21.0-23.9	23.9
13	13.6	13.6-21.1	21.1-24.3	24.3	14.0	14.0-21.6	21.6-24.6	24.6
14	14.3	14.3-21.4	21.4-24.2	24.2	14.7	14.7-22.1	22.1-24.9	24.9
15	15.2	15.1-22.0	22.0-25.0	25.0	15.5	15.5-22.5	22.5-25.3	25.3
16	15.6	15.6-22.4	22.4-25.0	25.0	16.0	16.0-22.6	22.6-25.4	25.4
17	16.1	16.1-22.7	22.7-25.2	25.2	16.1	16.1-23.0	23.0-25.9	25.9
18	16.4	16.4-23.0	23.0-25.7	25.7	16.4	16.4-23.1	23.1-26.0	26.0
